# Clinical Outcomes of Isobar TTL System with Isthmic Bone Grafting and Pedicle Screw‐Vertebral Plate Hook with Direct Repair of Defect for Lumbar Spondylolysis: A Matched‐Pair Case Control Study

**DOI:** 10.1111/os.13837

**Published:** 2023-08-14

**Authors:** Qiujiang Li, Bowen Hu, Zhuang Zhang, Qingquan Kong, Quan Gong, Limin Liu, Huiliang Yang, Lei Wang, Yueming Song

**Affiliations:** ^1^ Department of Orthopaedics, Orthopaedic Research Institute, West China Hospital Sichuan University Chengdu China

**Keywords:** Lumbar Spondylolysis, Dynamic Fixation, Direct Repair, Isobar TTL System, Vertebral Plate Hook

## Abstract

**Objective:**

Although direct isthmic repair, such as PSVPH, did not affect the mobility of the fixed segment and adjacent segment, it has a relatively low rate of isthmic fusion compared with conventional fusion. The Isobar TTL dynamic internal fixation system has been widely used in clinical practice and has achieved satisfactory clinical results. However, the use of the Isobar TTL system in combination with direct isthmic repair for lumbar spondylolysis has rarely been reported. The aim of this study was to compare the clinical and radiologic outcomes between patients who underwent Isobar TTL system and PSVPH with direct repair of defect for lumbar spondylolysis.

**Methods:**

Stepwise propensity score matching (PSM) for age and sex were performed to keep comparable clinical data between groups in this retrospective and matched‐pair case control study. A total of 50 patients diagnosed with lumbar spondylolysis underwent surgical implantation of the Isobar TTL group (*n* = 25) or PSVPH group (*n* = 25) from June 2009 to June 2016. Clinical outcomes were assessed using the Oswestry disability index (ODI), and visual analog score (VAS). Radiographic evaluations included range of motion (ROM) and the disc heights of stabilized segment and adjacent segment, adjacent segment degeneration (ASD) and bony fusion. Three‐dimensional reconstruction of lumbar CT scan was obtained to evaluate bone fusion of the isthmic at final follow‐up. The independent Student's *t* test and chi‐square test were applied to compare the differences between groups.

**Results:**

A total of 25 patients from TTL group were matched to 25 patients in PSVPH group for age, sex, body mass index (BMI), defect side, spondylolisthesis meyerding, and follow‐up duration. The intervertebral space height (IH) of stabilized segment at postoperative 1 week and final follow‐up in the TTL group was higher than those in the PSVPH group, respectively (*P* = 0.030; *P* = 0.013). The ROM of stabilized segment at final follow‐up in the TTL group was significantly lower than that in the PSVPH group (*P* < 0.001). The bony fusion rate at the final follow‐up was 88.0% (22/25 cages) in the TTL group and 80.0% (20/25 cages) in the PSVPH group. The ODI score at final follow‐up in the TTL group was significantly lower than that in the PSVPH group (*P* = 0.007).

**Conclusion:**

Overall, our data suggest that the Isobar TTL system outcomes are comparable to those in the PSVPH, with a similar high bony fusion rate as PSVPH, especially its wider indications as a new surgery.

## Graphical abstract

The findings of this study suggest that the Isobar TTL system outcomes are comparable to those in the PSVPH, with a similar high bony fusion rate as PSVPH, especially its wider indications as a new surgery.

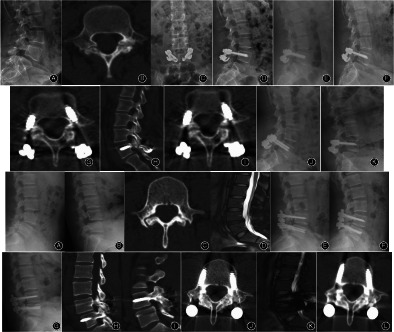

## Introduction

Lumbar spondylolysis is one of the more common causes of lower back pain in young patients.^1^, ^2^, ^3^ In some cases, non‐operative treatment, such as rest, physical therapy, pain management, and bracing, can effectively manage the symptoms of early lumbar spondylolysis.^4^ However, some patients who are resistant to conservative management or have increased vertebral slip usually require surgery.^5^, ^6^, ^7^, ^8^

Traditional fusion techniques join two or more vertebral bones together to provide stability to the affected area of the spine. This can result in the loss of mobility at the fused level and can increase the stress on the adjacent levels, potentially accelerating their degeneration.^9^, ^10^, ^11^, ^12^ In comparison, direct repair of the defect in the spine has some advantages compared to traditional fusion techniques. Direct repair techniques aim to preserve the anatomy and mobility of the affected segment of the spine, rather than immobilizing it.^13^, ^14^ This can help to maintain the normal motion and function of the spine, reducing the stress and strain on the adjacent levels and potentially slowing down their degeneration. One common direct isthmic repair technique is the pedicle screw‐vertebral plate hook (PSVPH) method, where a vertebral plate hook is attached to the pedicle screws that are placed into the vertebral body.^15^, ^16^ The plate hook helps to provide stability to the affected segment and reduce the load on the pars interarticularis, helping to alleviate pain and prevent further injury. Although direct isthmic repair, such as PSVPH, did not affect the mobility of the fixed segment and adjacent segment, it has a relatively low rate of isthmic fusion compared with conventional fusion. Lee et al.^13^ firstly described the short‐term clinical results of the direct repair with screw fixation for young patients with lumbar spondylolysis, only 25 patients (53%) were satisfied with direct repair surgery and the union rate of the pars defect was only 55% (26/47). Meanwhile, it is important to note that direct repair techniques are not suitable for all patients.^17^, ^18^, ^19^, ^20^ Postoperative outcomes are often poor in patients with significant disc degeneration. The adjacent‐level disc degeneration can cause new or worsening back pain, and in some cases, may require additional surgery. This is why alternative surgical techniques, such as dynamic stabilization systems like the Isobar TTL, have been developed.

The Isobar TTL dynamic internal fixation system consists of a polyethylene bearing and a titanium rod that are implanted in the spine to replace the damaged or degenerated disc.^21^, ^22^ The semi‐rigid design of the system allows for provided stability to the affected level of the spine and limited motion at the level of fixation, which helps to distribute the load more evenly and reduce the stress on the adjacent levels. By reducing the load on the discs and facet joints, it is thought to slow down the degeneration of adjacent levels, compared to traditional fusion techniques.^23^, ^24^ Huang et al.^25^ reported the Isobar TTL dynamic internal fixation system can prevent or delay the degeneration of intervertebral discs with a follow‐up of 48 months. However, there are few relevant studies and analyses about the Isobar TTL system in the treatment of lumbar spondylolysis. Only one previous study conducted by Wang et al. investigated the bony fusion of pars, which was confirmed on computed tomography (CT) scan, and significant pain relief was achieved in only 13 patients at postoperative 2 years.^26^ There is still a lack of current studies with large numbers of patients and adequate comparison about the Isobar TTL system in the treatment of lumbar spondylolysis.

The purposes of this study were (i) to compare the clinical and radiologic outcomes between patients who underwent Isobar TTL system and PSVPH with direct repair of defect for lumbar spondylolysis, and (ii) to assess whether the chosen technique can achieve satisfactory clinical outcomes and be maintained over time during follow‐up.

## Materials and Methods

This study reviewed patients with lumbar spondylolysis who underwent the Isobar TTL system with isthmic bone grafting or pedicle screw‐vertebral plate hook with direct repair of defect in our hospital from June 2009 to June 2016. This study was approved by the ethics committee of the West China Hospital. Written informed consent was obtained from all participants.

Inclusion criteria were as follows: (1) diagnosis of lumbar spondylolysis confirmed by imaging and physical examination; (2) bilateral lumbar spondylolysis with or without not more than grade 1 spondylolisthesis (Meyerding classification); (3) failed conservative treatment (at least 6 months); (4) age ≦ 30 years old.

Exclusion criteria were as follows: (1) patients with obvious contraindications to surgery; (2) patients with tumors, tuberculosis, or infections; (3) patients lacking complete clinical follow‐up data more than 2 years after surgery; (4) patients with preoperative adjacent segmental disc degeneration (Kellgren–Lawrence grade >2 or Pfirrmann grade >2). To eliminate the selective bias, we performed a 1:1 matching pair according to their age, sex, follow‐up, and radiographic features. Finally, there were 25 well‐balanced pairs of patients after PSM between Isobar TTL system and PSVPH.

### Surgical Procedures

All the patients underwent routine preoperative examination, including static and lateral flexion/extension X‐ray, CT scan, and magnetic resonance imaging (MRI). The surgery procedures were performed by one of four senior orthopaedic spine surgeons with more than 20 years of experience.

### Isobar TTL System

The patient was placed in the prone position, and the lumbar bridge was adjusted to reduce the anterior lumbar arch. After making the incision, the skin, subcutaneous tissue, and dorsal fascia were dissected to expose the spinal elements. Exposed and protected the bilateral facet joints of the upper and lower surgical level. The isthmic defect and surrounding lamina in the segment are exposed and protected, and the fibrous tissue and cartilage tissue are removed. The isthmic defect end is then ground down until a fresh bone surface is visible, creating a gap of about 3 mm. The harvested autologous bone (from the posterior superior iliac spine) is used to fill the defect and improve the union rate at the defect. After ensuring that there was no active bleeding, the drainage tube was placed routinely, and then the incision was closed. The drainage tube was removed within 72 h after surgery. Isobar TTL system: The pedicle screws were then implanted and positioned, and the Isobar TTL rods were inserted and connected to the screws. The nuts were then locked and stabilized to provide stability to the spinal column. PSVPH: The appropriate pedicle screws were selected and inserted into the vertebral body to provide stability, while the joint hook is attached to the screw and holds the broken end of the isthmus in place.

### Clinical Assessment

All patient‐related information is obtained from medical records. Clinical information, including age, sex, body mass index (BMI), smoking, defect level, defect side, follow‐up time, and surgery‐related complications, was collected preoperatively. Additionally, low back pain and neurological status were assessed using the visual analog scale (VAS) and Oswestry disability index (ODI) preoperatively, at 1‐week, 3‐month, final follow‐up postoperatively. All patients’ follow‐up information was collected during outpatient visits or telephone follow‐up. Patient satisfaction was evaluated using a questionnaire from a previous article.

### Radiographic Measurements

Static and lateral flexion/extension X‐ray were conducted to assess the radiological parameters: segmental range of motion (ROM); intervertebral space height (IH) of stabilized segments and the upper adjacent segments. The measurement of IH and ROM were performed preoperatively, at 1 week, and at final follow‐up after surgery. IH: the mean of anterior, middle, and posterior intervertebral space height. ROM: difference between the segmental angulations in flexion and extension X‐ray.

### Bony Fusion

The presence of trabeculation (the pattern of interconnecting bone struts) and bone bridging between cages and adjacent endplates, along with the absence of excessive translational motion (<3 mm) and angular motion (<5°), are indicators that fusion has occurred. In addition, the absence of a radiolucent gap (a region of low density on an X‐ray image) between the cages and endplates is also a sign of successful fusion.^27^ If there is uncertainty about the presence of bony fusion, a CT scan can be performed to confirm the formation of trabeculation between the bone autograft inside the strut and the adjacent endplates.

### Adjacent Segment Degeneration

A clinical diagnosis of adjacent segment disease (ASD) was made when X‐ray and MRI imaging showed one or more of the following signs at the adjacent segment^28^: (1) a decrease in intervertebral space height of more than 3 mm on anteroposterior X‐rays; (2) progression of anterior or posterior displacement of the vertebrae greater than 3 mm on lateral X‐rays; (3) sagittal translation greater than 3 mm or intervertebral angle change greater than 10° on lateral flexion/extension X‐rays; (4) progression of disc degeneration based on the Kellgren–Lawrence classification of greater than or equal to one grade. Although the MRI‐based disc grading system is a reliable tool for assessing disc degeneration,^29^ we mainly assessed postoperative disc degeneration degree based on X‐rays due to a very small number of MRI examinations being performed during long‐term follow‐up after surgery. Because ASD often occurs in the upper disc, only the upper disc degeneration was analyzed in this study.

### Statistical Analysis

For continuous variables, Student's *t* test was applied to compare the differences if two group data conformed to the normal distribution, otherwise Wilcoxon rank‐sum test was used. Categorical data were shown as percentages and comparisons between groups were analyzed by Chi‐square test or Fisher exact test. All statistical analyses were performed using SPSS 26.0 (SPSS Inc., Chicago, IL) and *P* values <0.05 were considered statistically significant.

## Results

### Demographic Data and Clinical Characteristics after PSM

At the end of the PSM analysis, 25 patients from TTL group were matched to 25 patients in the PSVPH group. The mean age for the whole group was 24.00 ± 5.97 years old, 34 (68.0%) were male and 16 (32.0%) were female. Mean (SD) follow‐up in the whole group was 56.76 (14.60) years. After PSM, all demographic data and clinical characteristics were similar between groups (*P* > 0.05) (Table 1).

**Table 1:** Patient demographic data and clinical characteristics between the two groups

Variables	Whole group (*n* = 50)	TTL group (*n* = 25)	PSVPH group (*n* = 25)	*t/X* ^2^ value	*P* value
Age, years	24.00 ± 5.97	23.96 ± 6.53	24.04 ± 5.50	−0.047	0.963
Sex, *n*(%)				0.368	0.544
Female	16 (32.0)	9 (36.0)	7 (28.0)		
Male	34 (68.0)	16 (64.0)	18 (72.0)		
BMI, kg/m^2^	23.38 ± 4.88	23.96 ± 4.56	22.80 ± 5.20	0.841	0.404
Smoking, *n*(%)				0.117	0.733
Yes	11 (22.0)	5 (20.0)	6 (24.0)		
No	39 (78.0)	20 (80.0)	19 (76.0)		
Defect side, *n*(%)				1.495	0.221
Unilateral	7 (14.0)	2 (8.0)	5 (20.0)		
Bilateral	43 (86.0)	23 (92.0)	20 (80.0)		
Defect level, *n*(%)				0.764	0.382
L4	19 (38.0)	11 (44.0)	8 (32.0)		
L5	31 (62.0)	14 (56.0)	17 (68.0)		
Spondylolisthesis Meyerding, *n*(%)				0.857	0.355
0	35 (70.0)	19 (76.0)	16 (64.0)		
1	15 (30.0)	6 (24.0)	9 (36.0)		
Follow up duration, months	56.76 ± 14.60	58.52 ± 15.39	55.00 ± 13.86	0.850	0.400

### Radiographic Outcomes

There was a mean IH of stabilized segment increase from 10.96 ± 1.42 mm preoperatively to 12.01 ± 1.58 mm postoperatively in the TTL group versus from 10.95 ± 1.16 mm preoperatively to 11.11 ± 1.26 mm postoperatively in the PSVPH group. Then, the mean IH slightly decreased at the final follow‐up. The IH of stabilized segment at postoperative 1 week and final follow‐up in the TTL group was higher than those in the PSVPH group, respectively (*P* = 0.030; *P* = 0.013). The ROM of stabilized segment at final follow‐up in the TTL group was significantly lower than that in the PSVPH group (*P* < 0.001). The bony fusion rate at the final follow‐up was 88.0% (22/25 cages) in the TTL group and 80.0% (20/25 cages) in the PSVPH group. At the final follow‐up, the ASD rates were 16.0% in the TTL group and 12.0% in the PSVPH group. No significant difference was observed in the ASD rate and bony fusion rate between the two groups (Table 2). In the TTL group, three patients did not achieve bony fusion after operation, and of which two patients had relief of symptoms after operation and received conservative treatment, of which one patient underwent revision surgery due to unilateral screw breakage. In the PSVPH group, five patients did not achieve bony fusion after operation, and of which three patients had relief of symptoms after operation and received conservative treatment, of which two patients underwent revision surgery.

**Table 2:** Summary of radiographic measurements between the two groups

	TTL group (*n* = 25)	PSVPH group (*n* = 25)	*t/X* ^2^ value	*P* value
IH of stabilized segment, mm				
Preoperative	10.96 ± 1.42	10.95 ± 1.16	0.045	0.964
Postoperative 1w	12.01 ± 1.58	11.11 ± 1.26	2.235	0.030
Final follow‐up	11.82 ± 1.62	10.78 ± 1.22	2.574	0.013
IH of adjacent segment, mm				
Preoperative	10.94 ± 1.26	10.98 ± 1.17	−0.120	0.905
Postoperative 1w	10.53 ± 1.30	10.76 ± 1.03	−0.688	0.495
Final follow‐up	10.65 ± 1.46	10.82 ± 1.19	−0.445	0.658
ROM of stabilized segment, °				
Preoperative	13.26 ± 3.85	13.44 ± 3.99	−0.166	0.869
Final follow‐up	4.58 ± 1.05	7.12 ± 1.79	−6.095	<0.001
ROM of adjacent segment, °				
Preoperative	3.33 ± 1.18	3.27 ± 1.22	0.177	0.860
Final follow‐up	3.52 ± 0.94	3.40 ± 0.92	0.455	0.651
ASD*, *n*(%)			0.166	0.684
Yes	4 (16.0)	3 (12.0)		
No	21 (84.0)	22 (88.0)		
Bony fusion*, *n*(%)			0.595	0.440
Yes	22 (88.0)	20 (80.0)		
No	3 (12.0)	5 (20.0)		

* At final follow‐up.

### Clinical Outcomes

**Figure 1:**
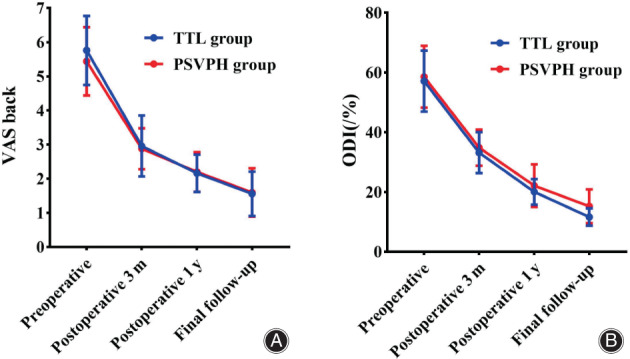
Longitudinal data describing the patient‐reported outcome measures visual analog score (VAS) back (A), and Oswestry disability index (ODI) (B) obtained preoperatively and during routine follow‐up postoperatively.

**Figure 2:**
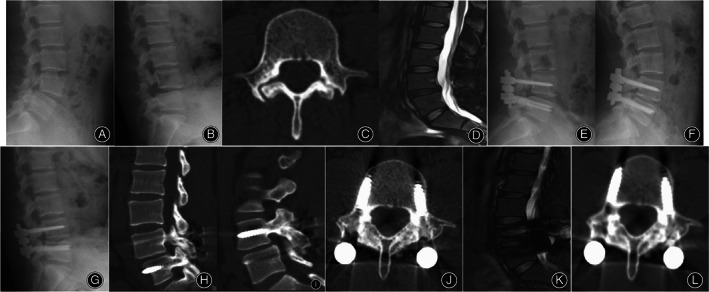
A 33‐year‐old female who underwent Isobar TTL dynamic fixation System with isthmic bone grafting for L4 bilateral isthmus.

**Figure 3:**
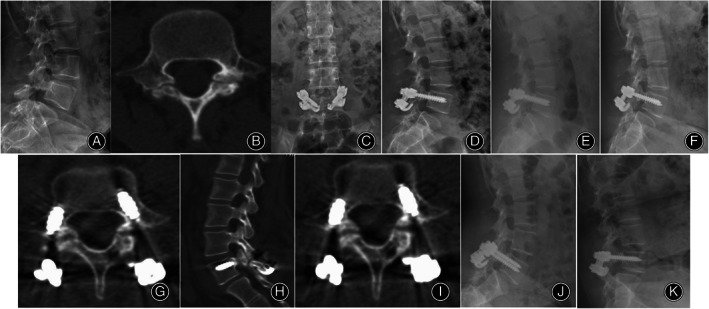
A 24‐year‐old female who underwent pedicle screw‐vertebral plate hook with direct repair of defect for L5 bilateral isthmus.

The VAS back and ODI score significantly improved in both groups at 3‐months, 1‐year postoperative and at final follow‐up. The ODI score at final follow‐up in the TTL group was significantly lower than that in the PSVPH group (*P* = 0.007). However, there were no significant differences in the VAS back score at any time point between the two groups preoperatively (*P* > 0.05) (Table 3) (Figure 1). There were also no cases of cerebrospinal fluid leakage, nerve injury, and postoperative infection complications. Figures 2 and 3 showed the representative cases for the TTL group and the PSVPH group, respectively.

**Table 3:** Visual analog score (VAS) and Oswestry disability index (ODI) scores between the two groups

	TTL group (*n* = 25)	PSVPH group (*n* = 25)	*t* value	*P* value
VAS back				
Preoperative	5.76 ± 1.01	5.44 ± 1.00	1.123	0.267
Postoperative 3m	2.96 ± 0.89	2.88 ± 0.60	0.373	0.711
Postoperative 1y	2.16 ± 0.55	2.20 ± 0.58	−0.250	0.804
Final follow‐up	1.56 ± 0.65	1.60 ± 0.71	−0.208	0.836
ODI, %				
Preoperative	57.12 ± 10.22	58.60 ± 10.33	−0.509	0.613
Postoperative 3m	33.20 ± 6.86	34.88 ± 6.04	−0.919	0.363
Postoperative 1y	20.04 ± 4.30	22.16 ± 7.15	−1.271	0.211
Final follow‐up	11.64 ± 2.90	15.24 ± 5.64	0.002	0.007

## Discussion

Lumbar spondylolysis is a common cause of lower back pain in young people.^4^, ^5^ Currently, due to a lack of relevant studies, there is no consensus on the optimal treatment approach for lumbar spondylolysis in young patients.Traditional lumbar fusion surgery restores spinal stability by fixing the vertebral body and pedicle, but it is often unsuitable for young patients, especially those with spondylolysis. Compared to traditional fusion techniques, direct repair technology and Isobar TTL dynamic fixation system can provide more stable support and better preserve the patient's biomechanical stability. In the present study, we found that intrasegmental isthmic bone graft can repair spondylolysis directly and restore that integrity of the lumbar isthmic anatomy, the Isobar TTL dynamic stabilization in the adjacent segment can provide a stable environment for bone grafting fusion in the pars defect area and maintain immediate stability after surgery. Meanwhile, the certain degree of micro‐motion can ensure a good fusion rate of isthmic bone graft and delay the degeneration of adjacent segments. Isobar TTL System with wider indications as a new surgery.

### Application of Direct Repair of the Isthmus in Lumbar Spondylolysis

Direct repair of the isthmus was first proposed by Japanese scholar Kimura^30^ in 1968 as a segmental repair technique. Kimura and colleagues attempted simple pars repair without fixation, restricting patients to bed rest for 2 months while wearing a plaster cast until bony fusion occurred. However, studies have shown a high incidence of pseudoarthrosis after surgery, and patients were required to remain in bed for long periods while wearing the plaster cast, which not only affected their daily activities but also compromised the integrity of their skin. Meanwhile, direct repair technology may not be a suitable option for patients with severe spinal slippage. Therefore, many scholars have supplemented the direct repair of the pars with internal fixation techniques such as Buck screw fixation,^31^, ^32^, ^33^ Scott transverse process‐spinous process wire ligation technique,^34^, ^35^, ^36^ and Morscher vertebral plate hook technique.^36^, ^37^ Lee et al.^13^ suggest that direct repair with screw fixation in young patients with lumbar spondylolysis may produce unsatisfactory outcomes at 1 year after surgery due to the low union rate (55%), which was lower than expected. Mohammed et al.^36^ reported a meta‐analysis showing that direct pars repair using pedicle screws is considered the optimal surgical option for the treatment of spondylolysis and low‐grade spondylolisthesis, with the highest fusion rates and lowest incidence of complications, compared to other techniques such as Buck repair,^31^, ^32^, ^33^ Morscher,^36^, ^37^ and Scott repair.^34^, ^35^, ^36^ However, the relative indications for direct repair are relatively strict, and it only fixes the posterior attachments or lamina, with limited fixation strength. As a result, there may be excessive motion in the surgical segment, and the bone fusion rate is relatively low. Mohammed et al.^36^ reported a fusion rate of 90.21% and a complication rate of 12.8% for pedicle screw‐based direct pars repair with fusion. Li et al.^38^ designed a new anatomic hook‐rod‐pedicle screw system for 15 young patients with lumbar spondylolysis and showed bilateral isthmus bone fusion in 14 cases and unilateral isthmus bone fusion in one case. We found in the present study that the bone fusion rate with PVSH was 80%, which was somewhat lower than in previous results. For young patients with mild spondylolisthesis, direct repair may show better results. In our study, there were more grade 1 spondylolisthesis patients with a mean age of 24 years. This could also be one of the reasons why the fusion rate in this study is slightly lower than the fusion rates reported in some literature.

### Limitations of PSVPH with Direct Repair of Defect

Some studies have reported that the lamina, hook screws, and pedicle screws are not on the same plane, making it difficult to implant lamina hooks in patients with abnormal lamina development.^26^, ^38^ Prior to installing the laminar hook, the spinous process and the muscles surrounding the lamina need to be separated, causing severe tissue damage. This is one reason for the fact that the PSVPH group has higher ODI score than TTL group. Furthermore, during the implantation process and follow‐up, it may cause lamina, transverse process, and spinous process fractures, shortening of the diseased isthmus, and disrupt the biting relationship between the lower articular process of the diseased vertebra and the upper articular process of the lower vertebra, leading to pain. In this study, eight patients had congenital lamina dysplasia, but the lamina hook system was successfully implanted in all cases. Therefore, congenital lamina dysplasia is not a significant factor limiting the use of lamina hooks.

### Application of Isobar TTL in Lumbar Spondylolysis

Isobar TTL is a type of posterior dynamic fixation system for the lumbar spine, which involves implanting a flexible Isobar rod within the intervertebral space and fusing it with the vertebral arch.^39^, ^40^ This allows for dynamic fixation of the lumbar spine, which is different from traditional fusion techniques that can limit normal vertebral motion. Isobar TTL is designed to preserve intervertebral disc and vertebral body motion, and reduce stress and strain on adjacent levels, thus reducing the risk of degeneration. Isobar TTL has shown good clinical outcomes in treating degenerative diseases of the lumbar spine, and it has certain advantages in terms of easy surgical operation and quick recovery.^41^, ^42^ However, there is currently little research on the application of Isobar TTL in patients with lumbar spondylolysis. Zeng et al.^39^ reported satisfactory fusion rate (88.5%) and slowed adjacent segment degeneration after lumbar fusion surgery using the Isobar TTL dynamic stabilization with pars bone grafting technique to treat lumbar spondylolysis with or without degree I slipping, indicating the safety and effectiveness of this approach. Xing et al.^26^ evaluated a new surgical technique, Isobar TTL dynamic stabilization with direct pars repair using wiltse approach, for the treatment of patients with spondylolysis with or without slight spondylolisthesis. All 13 patients with bony fusion of pars confirmed by CT scan at postoperative 2 years. Our study found that the fusion rate in the TTL group was slightly higher compared to the pedicle screw and hook group (88.0% vs. 80%). Direct repair of pars defect with bone grafting in the same segment can restore the integrity of the lumbar pars interarticularis. According to Wolff's law, fracture healing needs to increase the load on the fracture end, and mechanical stress stimulation is necessary to promote fracture healing and improve the quality of healing. Isobar TTL dynamic stabilization in the adjacent segment can provide a stable environment for bone grafting fusion in the pars defect area and maintain immediate stability after surgery, with a certain degree of micro‐motion. And so, ROM of stabilized segment at final follow‐up in the TTL group was lower than that in the PSVPH group (4.58 ± 1.05 vs. 7.12 ± 1.79). This approach can ensure a satisfactory fusion rate and slow down adjacent segment degeneration. The above factors collectively determine that the Isobar TTL will be an important method for repairing spondylolysis. This also explained why IH of stabilized segment at final follow‐up in the Isobar TTL group was higher than that in the PSVPH group.

### Isobar TTL Has Wider Indications as a New Surgery

Direct repair with laminar hooks may not be effective in addressing discogenic pain in patients with disc degeneration.^43^, ^44^, ^45^ As a result, this technique is usually not recommended for patients over 30 years old or those with severe disc degeneration based on preoperative MRI findings.^26^ The combination of isthmic bone graft repair and isobaric Isobar TTL stabilization can help to alleviate disc compression and overcome the limitations of laminar hooks. In our study with five patients with disc degeneration greater than grade 3, mild disc herniation, and mild spondylolisthesis, the Isobar TTL group achieved good results after long‐term follow‐up. MRI at the last follow‐up showed that the Isobar TTL had the advantage of delaying disc degeneration. Therefore, the Isobar TTL can achieve a similar high fusion rate and delay disc degeneration compared to laminar hooks, and has a wider indication and a relatively simple surgical procedure.

### Strengths and Limitations

However, several limitations still exist in our study. Firstly, this is a retrospective study in a single center, and small sample was another limitation for the study. Further prospective large‐scale randomized controlled trials are needed to confirm the current findings. Secondly, although MRI could provide a standardized assessment of disc status, MRI examination of every follow‐up is not routine. Third, the choice of the two different internal fixations for lumbar spondylolysis was not randomized, and the final results might be influenced by surgeon‐related factors to a certain degree.

### Conclusions

This retrospective study demonstrated that Isobar TTL dynamic fixation system can achieve the similar satisfactory radiographic and patient‐reported outcomes for lumbar spondylolysis as PSVPH. Attractively, Isobar TTL system with higher bony fusion rate and wider indications as a new surgery showed its great potential in the treatment of lumbar spondylolysis.

## Author Contributions

All authors had full access to the data in the study and take responsibility for the integrity of the data and the accuracy of the data analysis. Study concept and design: Q.L. and B.H. Acquisition of data: Q.L., B.H., Q.G., and Q.K. Analysis and interpretation of the data: Q.L. and B.H. Drafting of the manuscript: Q.L. and B.H. Critical revision of the manuscript for important intellectual content: Q.L., B.H., and H.Y. Statistical analysis: Q.L. and B.H. Obtained funding: H.Y. and Y.S. Study supervision: H.Y., L.W., and Y.S.

## Conflict of Interest Statement

The authors report no conflict of interest concerning the materials or methods used in this study or the findings specified in this paper.

## Ethics Statement

This study was performed in line with the principles of the Declaration of Helsinki. Approval was granted by the Ethics Committee of the West China Hospital (No. 2023‐178). Written informed consent was obtained from the parents.
